# Biocompatibility and biodistribution of functionalized carbon nano-onions (f-CNOs) in a vertebrate model

**DOI:** 10.1038/srep33923

**Published:** 2016-09-27

**Authors:** Marta d’ Amora, Marina Rodio, Juergen Bartelmess, Giuseppe Sancataldo, Rosaria Brescia, Francesca Cella Zanacchi, Alberto Diaspro, Silvia Giordani

**Affiliations:** 1 Optical Nanoscopy, Nanophysics, Istituto Italiano di Tecnologia, Via Morego 30, Genoa, 16163, Italy; 2 Nano Carbon Materials, Istituto Italiano di Tecnologia, Via Morego 30, Genoa, 16163, Italy; 3 Department of Computer Science, Bioengineering, Robotics and Systems Engineering, University of Genoa, Via Opera Pia 13, Genoa, 16145, Italy; 4 Nanochemistry Department, Istituto Italiano di Tecnologia, Via Morego 30, Genoa, 16163, Italy; 5 NIC@IIT, Istituto Italiano di Tecnologia, Via Morego 30, Genoa, 16163, Italy; 6 Department of Physics, University of Genoa, Via Dodecaneso 33, Genoa, 16145, Italy

## Abstract

Functionalized carbon nano-onions (f-CNOs) are of great interest as platforms for imaging, diagnostic and therapeutic applications due to their high cellular uptake and low cytotoxicity. To date, the toxicological effects of f-CNOs on vertebrates have not been reported. In this study, the possible biological impact of f-CNOs on zebrafish during development is investigated, evaluating different toxicity end-points such as the survival rate, hatching rate, and heart beat rate. Furthermore, a bio-distribution study of boron dipyrromethene (BODIPY) functionalized CNOs in zebrafish larvae is performed by utilizing inverted selective plane illumination microscopy (iSPIM), due to its intrinsic capability of allowing for fast 3D imaging. Our *in vivo* findings indicate that f-CNOs exhibit no toxicity, good biocompatibility (in the concentration range of 5–100 μg mL^−1^) and a homogenous biodistribution in zebrafish larvae.

Various carbon based nanomaterials are widely studied as promising imaging probes^1^,^2^,^3^, delivery vectors for drugs^4^,^5^,^6^ and as versatile theranostic nanomaterials^3^,^7^,^8^. Multi-shell fullerenes, known as carbon nano-onions (CNOs)^9^,^10^,^11^ are particularly attractive. They are sufficiently small (average diameters of 5 nm) to be carried in the circulatory system, have high cellular uptake, high biocompatibility and minimal systemic toxicity^12^. We have recently shown that CNOs decorated with water-soluble moieties (i) produce low inflammation *in vitro*, with significant reduction in the secretion of cytokines IL-1ß and (ii) decrease the recruitment of neutrophils and monocytes after injection in mice^12^. These important findings have made it possible to investigate fluorescent labelled CNOs as probes for high resolution imaging of intracellular trafficking and studies of biodistribution in different cell lines, including HeLa Kyoto cells^13^, MCF-7 human breast cancer cells^14^, HeLa cells^15^,^16^,^17^, and KB cells^17^. CNOs have been successfully applied in a variety of different fields including tribology^18^,^19^, catalysis^20^,^21^, sensing^22^, electronic applications^23^,^24^,^25^ and as molecular shuttles for fluorophores^13^,^14^,^15^,^16^,^26^.

For any nanomaterial utilized in an application that might lead to a release to the environment or to the exposure of living beings, in particular humans, an accurate risk assessment as well as a toxicological screening is highly needed^27^,^28^. The aquatic environment is of high importance since any contamination of water might lead to a wide distribution of the contaminant and thus to major pollution. All the biological *in vitro* studies investigating the effects of small CNOs (diameter of approx. 5 nm) with different surface functionalization on a variety of different cell cultures describe CNOs as a highly biocompatible nanomaterial. The effect of large CNOs (diameter of approx. 30 nm) on the immune system indicates that the cell response is highly dependent on the structure^29^. The inflammatory potential of small f-CNOs on immortalized bone-marrow-derived mouse macrophages and mouse bone-marrow-derived dendritic cells is found to be negligible and significantly lower than the effects of similarly functionalized single wall carbon nanotubes^12^. A recent report comparing large CNOs (diameter of 50–100 nm) with multiwall carbon nanotubes corroborates these findings^30^. We recently showed that small functionalized CNOs have no significant adverse effects on three weeks old freshwater polyp *Hydra vulgaris*^31^. The results reported on this very simple and basal animal, in addition to *in vitro* studies, suggest that CNO is a biocompatible and safe nanomaterial, but are not sufficient to rule out possible risks of CNOs exposure and release. Additional long-term toxicity studies of CNOs on complex organisms, as well as the fate of CNOs in biological systems, are strongly needed. Zebrafish represents an emerging and excellent model organism, mainly due to the fact that it has a remarkable similarity in the molecular signalling processes, cellular structure, anatomy and physiology to other higher order vertebrates^32^,^33^,^34^,^35^,^36^,^37^. Moreover, zebrafish embryos are ideal for high-throughput screening due to their external development, optical transparency, and short breeding cycle^38^,^39^. Zebrafish development represents a valuable tool to assess the *in vivo* toxicity and biocompatibility of drugs, chemicals and nanomaterials, with a focus on the developmental effects, and to obtain toxicity information at the whole animal level^40^,^41^,^42^,^43^,^44^,^45^. Several approaches using zebrafish embryos or larvae as an animal model have been developed in recent years to assess embryonic effects of chemicals, drugs and nanomaterials^46^,^47^,^48^, in order to predict the potential risk induced by the nanomaterial’s exposure on human health. We therefore decided to employ zebrafish as a vertebrate model to investigate the possible effects induced by benzoic acid functionalized CNOs (benz-CNOs, Fig. 1a left) and fluorescent boron dipyrromethene tagged CNOs (BODIPY-CNOs, Fig. 1a right). Different toxicological end-points such as the incidence of malformations, spontaneous movements and hatching rate/time disturbance are assessed during the zebrafish’s development. Moreover, the fluorescent BODIPY-CNOs biodistribution in zebrafish at completed organogenesis is studied using inverted selective plane illumination microscopy in order to probe their *in vivo* internalization inside the zebrafish larvae.

## Results and Discussion

### Preparation of CNOs

The preparation of benz-CNOs and BODIPY-CNOs follows a protocol reported by our group^14^. Briefly, the pristine CNOs are functionalized with benzoic acid moieties by reacting them in a dimethylformamide/water mixture with *in situ* generated diazonium salts. Subsequently, the benzoic acid groups are functionalized with a *meso*-phenol substituted BODIPY fluorophore in an esterification reaction. An average of 37 BODIPY molecules per CNO are attached following this synthetic procedure. The number of BODIPY molecules per CNO (made of 9 shells of graphitic carbon) is calculated based on TGA data as published earlier^14^, following the protocol of Prato *et al*.^49^.

The fluorescently tagged CNOs show an intense green fluorescence with an emission maximum at about 512 nm. The fluorescence quantum yield in DMSO is estimated to be about 0.17^14^.

Bright Field Transmission Electron Microscopy (BF-TEM) and High Resolution Transmission Electron Microscopy (HR-TEM) are used to characterize the benz-CNOs and BODIPY-CNOs. The presence of small agglomerates with a size lower than 100 nm is shown in both samples (Supplementary Information, Figure S1a and Figure S2a respectively).

**Figure 1:**
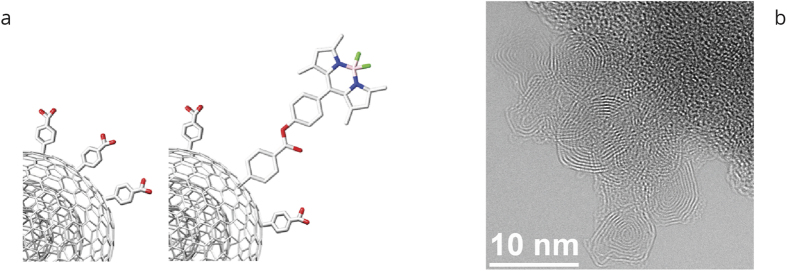
(**a**) Schematic representation of benz-CNOs (left) and BODIPY-CNOs (right); Blue, nitrogen atom (N); pink, boron atom (**B**); green, fluorine atom (F). (**b**) HR-TEM image of BODIPY-CNOs.

HR-TEM is used to highlight the CNO internal structure. A representative HR-TEM image of BODIPY-CNOs is illustrated in Fig. 1b, which clearly shows individual CNOs with a diameters of 7 nm, with 9 concentric graphitic shells (with measured intershell spacing of 3.4 Å). HRTEM images of individual benz-CNOs and BODIPY-CNOs are shown in Supplementary Information, Figure S1b and Figure S2b respectively. The height distributions analyses based on HRTEM images reveal a mean diameter of 6.8 and 7.1  for benz-CNOs and BODIPY-CNOs respectively (see Supplementary Information Figure S5).

Dynamic Light Scattering (DLS) and Z-potential measurements are performed in order to characterize the nanoparticles’ physicochemical properties, including size and surface electrostatic charge. DLS measurements are performed in embryo medium to mimic the conditions used in biological experiments. DLS measurements in deionized water are also performed as reference. Initially, benz-CNOs and BODIPY-CNOs are dissolved in deionized water at a mass concentration of 1.0 mL^−1^ and then diluted with embryo medium to a final mass concentration of 5, 10, 50 and 100 μg mL^−1^. Z-potential measurements are conducted on the same samples. Benz-CNOs have an hydrodynamic diameter located at around 190 nm in aqueous medium and between 210 and 350 nm in embryo medium (see Supplementary Information, Figure S3 and Table S1). Zeta potential values of −42 mV and −30 mV is obtained for benz-CNOs in water and embryo medium respectively (see Supplementary Information, Table S3). BODIPY-CNOs have hydrodynamic diameter between 100 and 170 nm in an aqueous medium and at around 210 in embryo medium (see Supplementary Information, Figure S4 and Table S2). Zeta potential values ranging from −23 mV to −36 mV and at around −30 mV is obtained for BODIPY-CNOs in water and embryo medium respectively (see Supplementary Information, Table S4). As previously reported by our group^14^, the values obtained for BODIPY-CNOs are less negative than the one found for benz-CNOs.

### Toxicity evaluation of benz-CNOs and BODIPY-CNOs

To elucidate the effects induced by benz-CNOs and BODIPY-CNOs on the development of zebrafish, CNO dispersions of different CNO mass concentrations (100, 50, 10, 5 μg mL^−1^) in the embryo growth medium are prepared by ultrasonic treatment for 30 min at 37 kHz. The embryos are exposed to the different concentrations of benz-CNOs and BODIPY-CNOs and the effects are investigated at different stages of development.

**Figure 2:**
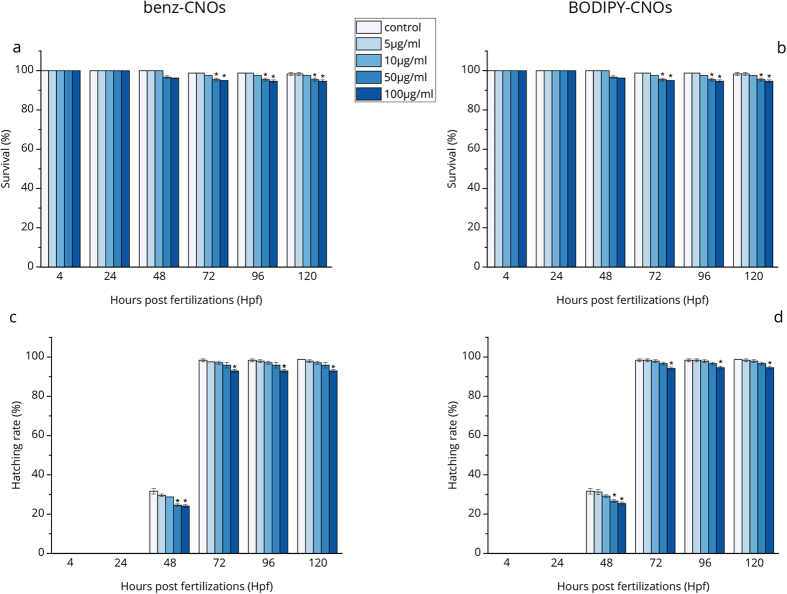
Survival rate of zebrafish embryos/larvae exposed to different concentrations of benz-CNOs (**a**) and BODIPY-CNOs (**b**) and hatching rate of zebrafish embryos/larvae exposed to different concentration of benz-CNOs (**c**) and BODIPY-CNOs (**d**). Data are calculated as means ± S.D., from three independent experiments, n = 80 (*p ≤ 0.01 compared to the control).

First, the survival and hatching rates of treated zebrafish embryo/larvae are measured at specified time points. As shown in Fig. 2a,b, up to 10 μg mL^−1^ of benz-CNOs and BODIPY-CNOs no considerable changes in the survival rate of exposed embryos/larvae are observed in comparison with that of the control groups, whereas a significant difference (p ≤ 0.01) is present at higher concentration of CNOs (50 and 100 μg mL^−1^) between the 72 and 120 hours post fertilization (hpf). Nevertheless, in the first 24 hpf no dead embryos are observed and at 72 hpf the survival rate is higher than 95% for both types of CNOs.

Moreover, as depicted in Fig. 2c,d, embryos of the treated group start to hatch between 48 and 72 hpf, that is the normal temporal window for the hatching^50^. Compared to the untreated control, both types of CNOs do not induce any embryonic developmental delay. Figure 2c,d show that at the highest CNO mass concentration (100 μg mL^−1^), the hatching rates are 92.9% and 94.2% for larvae of 72 hpf treated with the benz-CNOs and BODIPY-CNOs, respectively, even if with a significance difference compared with the negative control (p ≤ 0.01).

According to the OECD methodology^51^, to consider a nanomaterial as non-toxic, the survival and hatching rates of the embryos should be ≥ 90% and ≥80%, respectively. Consequently, our results reveal that benz-CNOs and BODIPY-CNOs have a concentration dependent behaviour on the survival and hatching rates but exert no detectable toxicity in zebrafish during the development.

**Figure 3:**
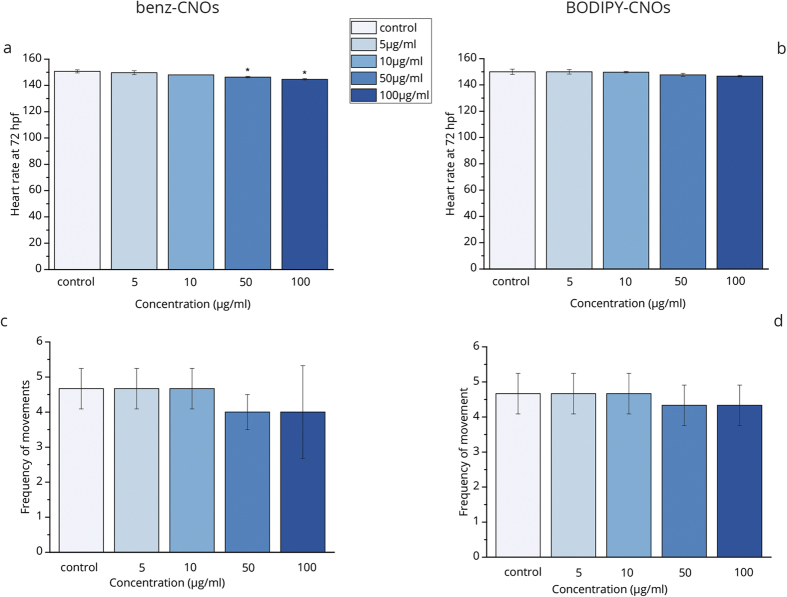
Heart beat rate of zebrafish larvae at 72 hpf exposed to different concentration of benz-CNOs (**a**) and BODIPY-CNOs (**b**) and frequency of voluntary movements of the larvae at 72 hpf exposed to different concentration of benz-CNOs (**c**) and BODIPY-CNOs (**d**). Data are calculated as means ± S.D., from three independent experiments, n = 80 (*p ≤ 0.01 compared to the control).

Furthermore, the heart beat rate and frequency of movements in zebrafish larvae at 72 hpf is monitored for abnormalities induced by the CNOs exposure. The number of heart beats for the larvae treated with benz-CNOs (144–149/min) and BODIPY-CNOs (146–150/min) is comparable to control group (150 beats/min) (Fig. 3a,b). The frequency of movements, as response to touching, is of 4–4.6 /min and of 4.3–4.6 /min for the ones treated with the benz-CNOs and BODIPY-CNOs, respectively, without change compared to that of the control (4.6 /min) (Fig. 3c,d).The results obtained show no significant adverse effects of CNOs on both the parameters.

**Figure 4:**
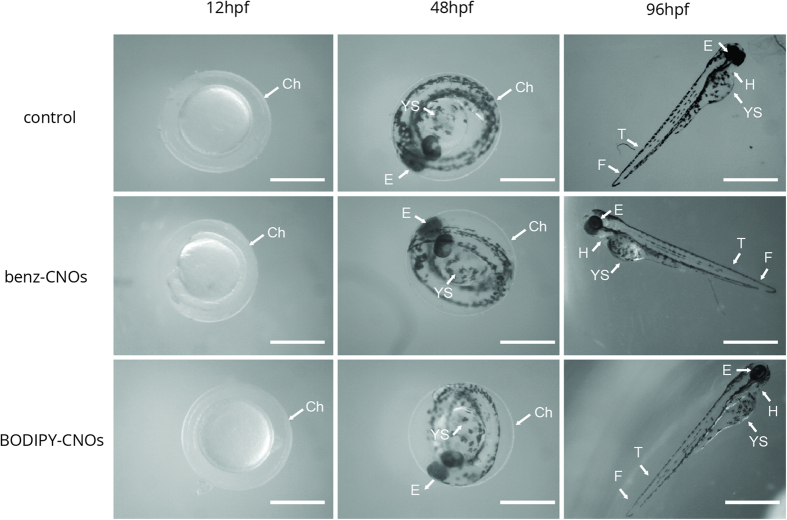
Representative optical images of zebrafish exposed to 0 (control) and 100 μg mL^−1^ of benz-CNOs and BODIPY-CNOs at 12, 48 and 96 hpf. Scale bars, 500 μm.Ch, chorion; E, eye; YS, yolk sac; H, heart; T, tail; F, finfold.

**Figure 5:**
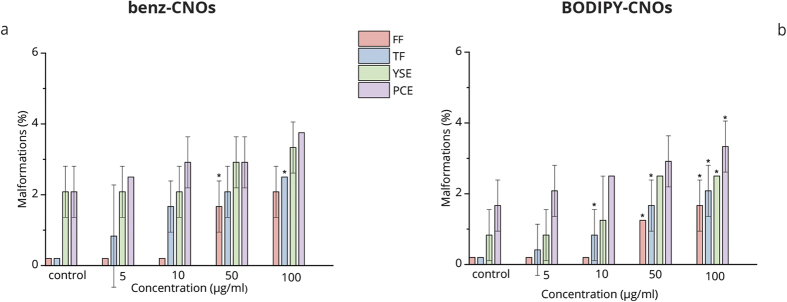
Histograms of the percentages of malformations on larvae with each type of abnormalities versus benz-CNOs (**a**) and BODIPY-CNOs (**b**) concentrations at 96 hpf. PCE, pericardial edema; YSE, yolk sac edema; TF, tail flexure; FF, fin fold abnormality. Data are expressed as means ± S.D., from three independent experiments, n = 80 (*p ≤ 0.01 compared to the control).

Finally, the effects of CNOs on the organogenesis of zebrafish are evaluated. The embryos exposed to different CNO mass concentrations grow normally into the larval stage without signs of possible toxicity. Figure 4 shows the biocompatibility of CNOs as observed by the morphological development. Embryos imaged at 12, 48 (see Supplementary Figure S6) and 96 (see Supplementary Figure S7) hpf, corresponding respectively to segmentation, hatching and larvae stages, show negligible malformation. The noted malformations are yolk sac edema (YSE), pericardial edema (PCE), fin fold abnormalities (FF) and tail flexure (TF). In the embryos/larvae exposed to 50 and 100 μg mL^−1^ of benz-CNOs, there is a significant difference (p ≥ 0.01), for the FF and TF respectively (as shown in Fig. 5). Considering the BODIPY-CNOs, we observe a significant difference at 10 (FF), 50 (FF and TF), and 100 μg mL^−1^ (TF, FF, YSE, PCE). Nevertheless, the total percentages of abnormalities, induced by CNOs, are less than 4% (representing a score of 1 on a published 4-point malformation scale^38^).

Our findings demonstrate the non-toxicity and good biocompatibility of CNOs on zebrafish during the development. These results are in good agreement with previous toxicological research performed *in vitro* on MCF-7 cells^14^ and *in vivo* on *Hydra vulgaris*^31^. In particular, in the freshwater polyp *Hydra*, it was demonstrated that shorter time (24 h) exposure of benz-CNOs on animal of three weeks old did not induce adverse effects in terms of change of morphology, behavior and reproductive capability^31^.

The results presented here are not in line with the current literature regarding other carbon nanomaterials probed on zebrafish. Recent studies reported that carbon nanomaterials induce dose and time-dependent toxicity in zebrafish during the development, with only one exception^52^. Exposure or injection of fullerene^53^,^54^, single-walled carbon nanotubes (SWCNTs)^55^, graphene quantum dots^56^, and graphene oxide^57^,^58^ lead to a high mortality rate, delayed development and different distinct deformations of the embryos such as pericardial/yolk sac edema, tail flexure, and head defects. As the single exception, Kang *et al*.^52^ reported a high biocompatibility and very low biotoxicity of carbon quantum dots (C-QDs) in zebrafish. Embryos soaked with C-QDs grow normally, presenting only a small amount of abnormalities, comparable to that of the negative control.

Noteworthy, the toxic and adverse response of zebrafish embryos is strongly dependent on the hydrophobicity and solubility of the different carbon nanomaterials, which can be strongly affected by a different functionalization of the surface. The zebrafish until 72 hpf is surrounded by a chorion that protects the embryos^59^. The nanomaterials are able to pass the chorion through the pores (0.6–0.7 μm), but a low solubility can lead the nanomaterials to agglomerate, thus preventing uptake by the embryos and therefore affecting the reliability of the toxicity studies.

In this framework, different reports on these nanomaterials being either non-toxic^59^ or toxic^55^, depend on the degree of agglomeration. Our benz-CNOs and BODIPY-CNOs do not seem to form big agglomerates, since we do not see any dark spots through the stereomicroscope on the chorion, and the surface is completely transparent (Fig. 4).

Here we report for the first time the *in vivo* non-toxicity and biosafety of benz-CNOs and BODIPY-CNOs on zebrafish during the development by evaluating different toxicological endpoints. No adverse effects of CNOs are observed and noted for zebrafish embryo/larvae even at the higher mass concentration tested (100 μg mL^−1^). Results presented herein clearly demonstrate that CNOs have a good and high biocompatibility making them suitable for biomedical applications. The results obtained here by investigating the consequences of CNOs exposure on zebrafish, can be easily utilized to predict the effects of these nanomaterials on other vertebrates, since there is a high similarity between zebrafish and humans during development in terms of genetic, physiological and cellular processes.

### Biodistribution of BODIPY-CNOs in zebrafish larvae

The biodistribution of BODIPY-CNOs is studied in a complete developed larvae (72 hpf). Previous works elucidating the biodistribution of different nanomaterials in cells and in different biological model systems, use a variety of imaging techniques such as Transmission Electron Microscopy (TEM) and fluorescence microscopy^60^,^61^. Specifically, fluorescence microscopy is a noninvasive imaging technique and provides useful insights into the physiological structure and function of living organisms at cellular and subcellular resolution and allows for the direct visualization of the underlying functions of physiological processes in living cells or tissues.

Our previous study^14^ demonstrated the suitability of CNOs for high resolution imaging as shown by confocal images of live MCF-7 cells treated with the BODIPY-CNOs.

In this work, the *in vivo* imaging of BODIPY-CNOs (100 μg mL^−1^) in zebrafish larvae is performed using, as advanced microscopy technique, the iSPIM.The positive features provided by iSPIM are dual. First this system works under less photo-toxic conditions than the scanning based techniques such as confocal microscopy, thanks to the reduced photon density delivered on the sample. Second, iSPIM allows for fast 3D imaging making this technique a powerful tool to investigate the biodistribution of fluorescent nanomaterials in zebrafish embryos/larvae^62^.

**Figure 6:**
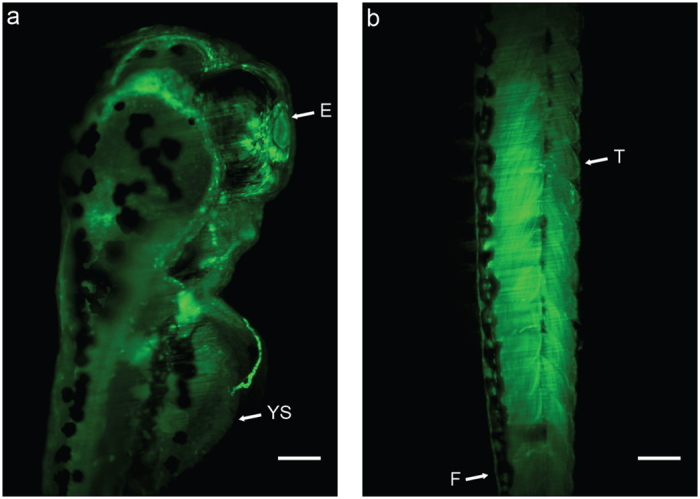
Maximum intensity projections of the superior part (**a**) and tail (**b**) of treated larvae (100 μg mL^-1^ of BODIPY-CNOs. Exposure time: 200 ms, step size: 0.7 μm. Scale bars, 100 μm. E, eye; YS, yolk sac; T, tail; F, finfold.

Figure 6a,b illustrate the *in vivo* distribution of green fluorescent CNOs in the zebrafish developed at complete organogenesis and exposed to 100 μg mL^−1^ mass concentrations of the BODIPY-CNO nanomaterial (z-stack images of Fig. 6a,b are reported as Supplementary Figure S8 and Figure S9). CNOs enter the embryos via chorion pores until 48–72 hpf by simply soaking of the embryos in the solution. After the hatching, zebrafish larvae took the CNOs up through both swallowing and skin-absorption^63^. As shown in Supplementary Figure S7, BODIPY-CNOs are present in the blood vessels, indicating that they are able to enter in the circulatory system and accumulate in different areas of the whole zebrafish. The BODIPY-CNOs exhibit a homogeneous distribution. In particular, Fig. 6a represents the 3D maximum projection of the upper part of the larvae, where the BODIPY-CNOs accumulate selectively in the head with the highest brightness at the retina level. This suggests a high affinity of CNOs for this tissue. Figure 6b reveals the green signal of the CNO immobilized BODIPY fluorophore throughout the whole trunk of the larvae. Such results indicate a ubiquitous distribution of the CNOs in the larvae’s body at 72 hpf.

## Methods

### CNOs Synthesis

Pristine CNOs were prepared by the thermal annealing of nanodiamonds following a previously reported procedure^64^. The procedures for the chemical functionalization of the pristine CNOs, as well as the spectroscopic, microscopic and physicochemical characterization of the functionalized CNOs, were described elsewhere^14^. For the biological experiments, CNO dispersions were prepared in aliquots of zebrafish embryo medium (*i.e.* NaCl, KCl, CaCl_2_ · 2H_2_O and MgCl_2_ · 6H_2_O; pH 7.2), by ultrasonication in a bath sonicator at 37 kHz for 30 min. Initially a dispersion with a CNO mass concentration of 100 μg mL^−1^ was prepared, which is then diluted to the desired mass concentration.

### Thermogravimetric analysis

TGA was conducted on a TA Q500 analyzer, using a Pt pan as sample holder. The measurement was performed in air using a heating rate of 10 °C/min. After equilibrating the sample at 30 °C for 5 min and then at 100 °C for additional 20 min, the sample weight was monitored until 900 °C had reached by the furnace.

### Transmission electron microscopy

For BF-TEM and HR-TEM imaging, all the CNO samples were suspended in spectroscopic grade ethanol, mildly sonicated for 3 min and deposited onto holey carbon film-coated Cu grids. BF-TEM imaging were performed on a FEI Tecnai G2 F20 TWIN TMP instrument equipped with a Schottky source operated at 200 kV. In order to increase the contrast, a 7.5 mrad objective aperture was used. For HR-TEM imaging, all the CNO samples were suspended in spectroscopic grade ethanol, mildly sonicated for 3 min and deposited onto ultrathin carbon film/holey carbon film-coated Cu grids. Energy-filtered HR-TEM imaging was carried out on a Jeol JEM-2200FS instrument equipped with a Schottky source operated at 200 kV, a CEOS image aberration corrector and an in-column energy filter (Ω-type). All the reported HR-TEM images were acquired by using a 5 eV energy-selecting slit in order to select elastically scattered electrons.

### DLS and Zeta-potential

DLS measurements were performed on the Malvern Nano-ZS instrument operating in backscattering (173°) mode and analyzed with the proprietary software Zetasizer, with automatic selection of the optimal detector position and number of independent measurements. Initially, benz-CNOs and BODIPY-CNOs were dissolved in deionized water at a concentration of 1.0 mg mL^−1^ and then diluted with embryo medium to a final concentration of 5, 10, 50 and 100 μg mL^−1^. Each solution was sonicated for 30 min at 37 Hz. Z potential measurements were performed on the same solutions, by means of the same apparatus using the disposable proprietary Z-potential cuvettes.

### Zebrafish husbandry

Wild-type (wt) adult zebrafish were purchased from a commercial source and were maintained in a circulating aquarium system on a 14 h light/10h dark cycle at 28 °C. The animals were fed three times daily with dry flake and were crossed as shown in the “Zebrafish Book”^50^.

### Embryo toxicity study

Healthy zebrafish eggs were collected at 4 hpf, washed in embryo medium and placed into 24-well culture plates. Embryos were incubated with different concentrations of benz-CNOs and BODIPY-CNOs (100, 50, 10, 5 μg mL^−1^) and with embryo medium as negative control at 28 °C until 120 hpf. The survival rate, hatching rate, heart beat rate, frequency of movements and possible presence of malformations were evaluated directly using a stereomicroscope (Stereo Discovery.V8, Zeiss Microscopy) attached to a CCD camera at different time points (12, 24, 48, 72, 96 and 120 hpf). To maintain an accuracy statistics, all experiments were performed three times. All animal experiments were performed in full compliance with the revised directive 2010/63/EU.

### Statistical Analysis

Data were expressed as mean ± S.D. and significance was determined by one-way analysis of variance (ANOVA) in combination with Holm-Sidak post hoc in order to compare each treatment group with the negative controls. A value of p ≤ 0.01 was considered statistically significant (marked by an asterisk in Figs 2, 3 and 5).

### Biodistribution of CNOs in the larvae

The *in vivo* biodistribution of BODIPY-CNOs in zebrafish larvae was performed by iSPIM. Larvae of 72hpf were selected after the exposure to 100 μg mL^−1^ BODIPY CNOs and anesthetized with 0.016% (w/v) tricaine (ethyl 3-aminobenzoate methanesulfonate) (Sigma-Aldrich).

### Inverted selective plane illumination microscopy (iSPIM)

Zebrafish imaging was carried out with a custom-built iSPIM microscope, mounted on a vertically oriented optical breadboard. The illumination unit was based on a conventional SPIM configuration in which a cylindrical lens (Thorlabs LJ1703RM, f = 75 mm) created the planar illumination focusing the light in the back focal plane of an objective lens (Nikon CFI Plan Fluor 10XW, 0.3 NA, 3.5 mm WD). A 488 nm laser beam (Obis Coherent) was used for the excitation. The signal detected from the detection objective (Nikon CFI Plan Fluor 10XW, 0.3 NA, 3.5 mm WD) was focused by a tube lens (Thorlabs AC254-200-A-ML) onto an sCMOS Camera (Hamamatsu Orca Flash 4.0) and filtered by a dichroic (Chroma 491 RDCXT) and emission filter (Semrock 525/50 nm). Zebrafish embryo was placed on a standard microscope petri dish (50 mm Sterilin Petri Dish, Thermo Scientific) filled with embryo medium and placed over a piezo-driven stage (Physik Instruments P-563.3CD) that allowed fine translation of the sample through the static light sheet to get sample 3D stack.

## Additional Information

**How to cite this article**: d′Amora, M. *et al*. Biocompatibility and biodistribution of functionalized carbon nano-onions (f-CNOs) in a vertebrate model. *Sci. Rep.* **6**, 33923; doi: 10.1038/srep33923 (2016).

## Supplementary Material

Supplementary Information
