# Supplementary material for: Biocompatibility and biodistribution of functionalized carbon nano-onions (f-CNOs) in a vertebrate model
Source: Sci Rep. 2016 Sep 27;6:33923. doi: 10.1038/srep33923 (PMC5037369; doi:10.1038/srep33923)

Biocompatibility and biodistribution of functionalized carbon nano-onions (f-CNOs) in a vertebrate model

Marta d'Amora[1], Marina Rodio[2], Juergen Bartelmess[2†], Giuseppe Sancataldo[1,3], Rosaria Brescia[4], Francesca Cella Zanacchi[1,5‡], Alberto Diaspro[1,5,6], Silvia Giordani[2*]

[1] Optical Nanoscopy, Nanophysics, Istituto Italiano di Tecnologia, Via Morego 30, Genoa, 16163, Italy.
[2] Nano Carbon Materials, Istituto Italiano di Tecnologia, Via Morego 30, Genoa, 16163, Italy.
[3] Department of Computer Science, Bioengineering, Robotics and Systems Engineering, University of Genoa, Via Opera Pia 13, Genoa, 16145, Italy.
[4] Nanochemistry Department, Istituto Italiano di Tecnologia, Via Morego 30, Genoa, 16163, Italy.
[5] NIC@IIT, Istituto Italiano di Tecnologia, Via Morego 30, Genoa, 16163, Italy.
[6] Department of Physics, University of Genoa, Via Dodecaneso 33, Genoa, 16145, Italy.

[†]Current address: Federal Institute of Materials Research and Testing (BAM), Division 1.9, Richard-Willstaedter-Str. 11, 12489 Berlin, Germany.
[‡]Current address: The Institute of Photonics Sciences (ICFO) Av. Carl Friedrich Gauss, 3, 08860 Castelldefels (Barcelona), Spain.

[*]Correspondance to Silvia Giordani

Supplementary Figures and Tables

Figure S1. ***Bright Field –Trasmission Electron Microscopy and High Resolution TEM analyses of benz-CNOs***

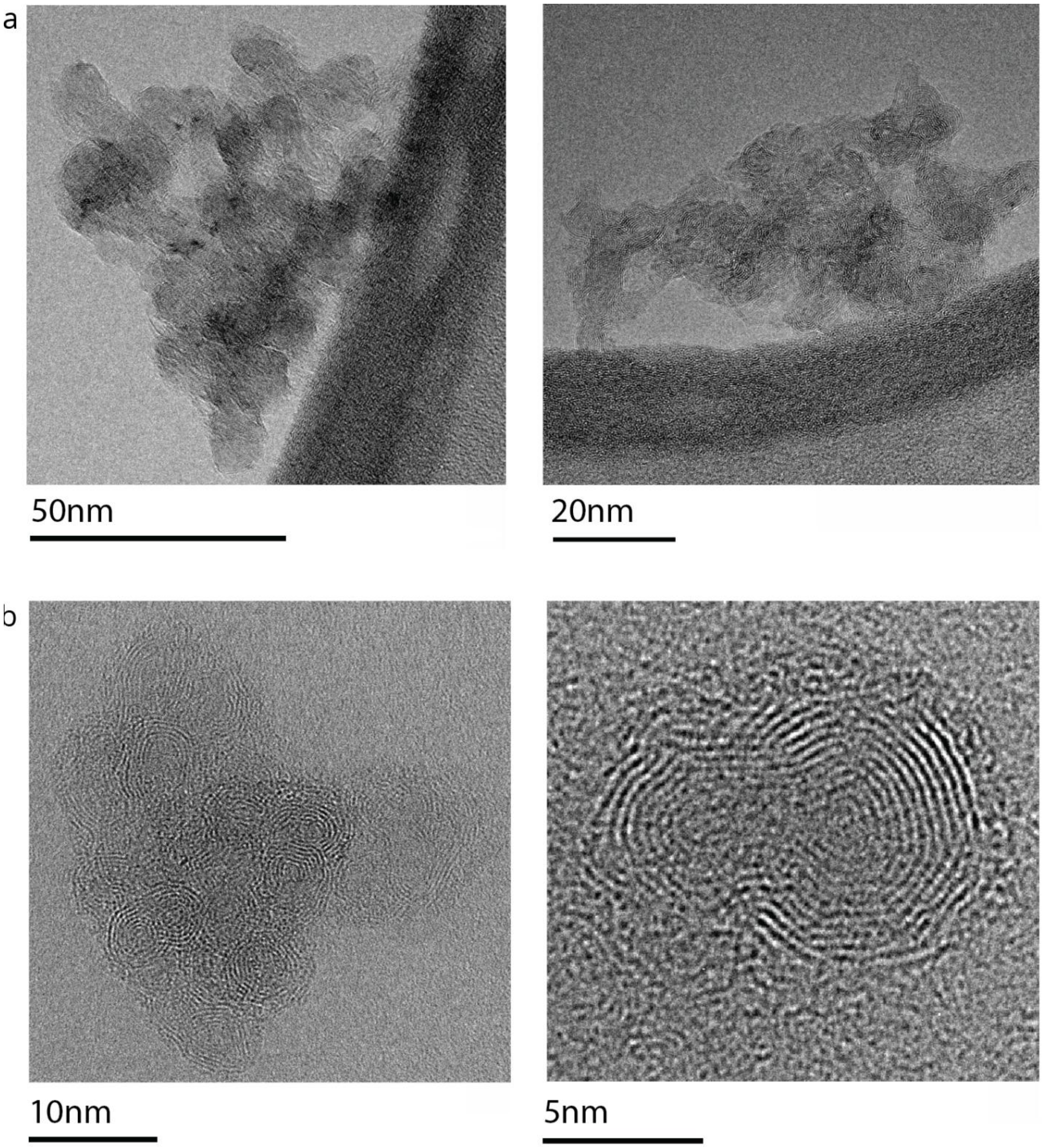

a) Overview HR-TEM images of benz-CNOs aggregates partially suspended on holes in the amorphous carbon film. b) Zero-loss filtered HR-TEM images of aggregates formed by a small number of benz-CNOs on an ultrathin amorphous carbon film. Noteworthy, panel b, right image, shows an individual benz-CNO.

Figure S2. ***Bright Field –Trasmission Electron Microscopy and High Resolution TEM analyses of BODIPY-CNOs***

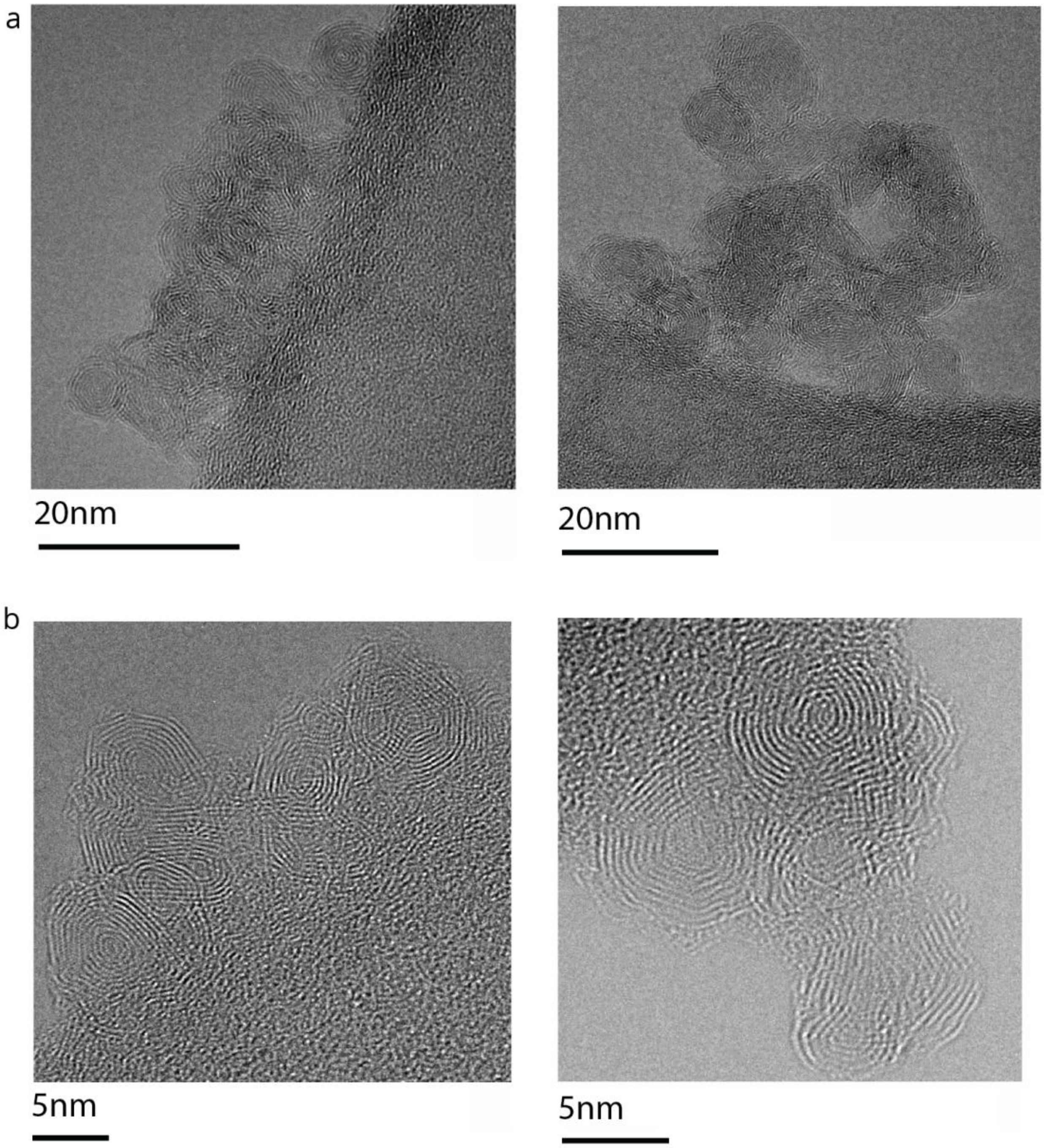

a) Overview HR-TEM images of BODIPY-CNOs aggregates partially suspended on holes in the amorphous carbon film. b) Zero-loss filtered HR-TEM images of aggregates formed by a small number of BODIPY-CNOs suspended on holes in the amorphous carbon film. The measured inter-shell spacing in all HR-TEM images is 3.4 Å.

Figure S3. ***Benz-CNOs diameter distribution obtained by DLS measurements***

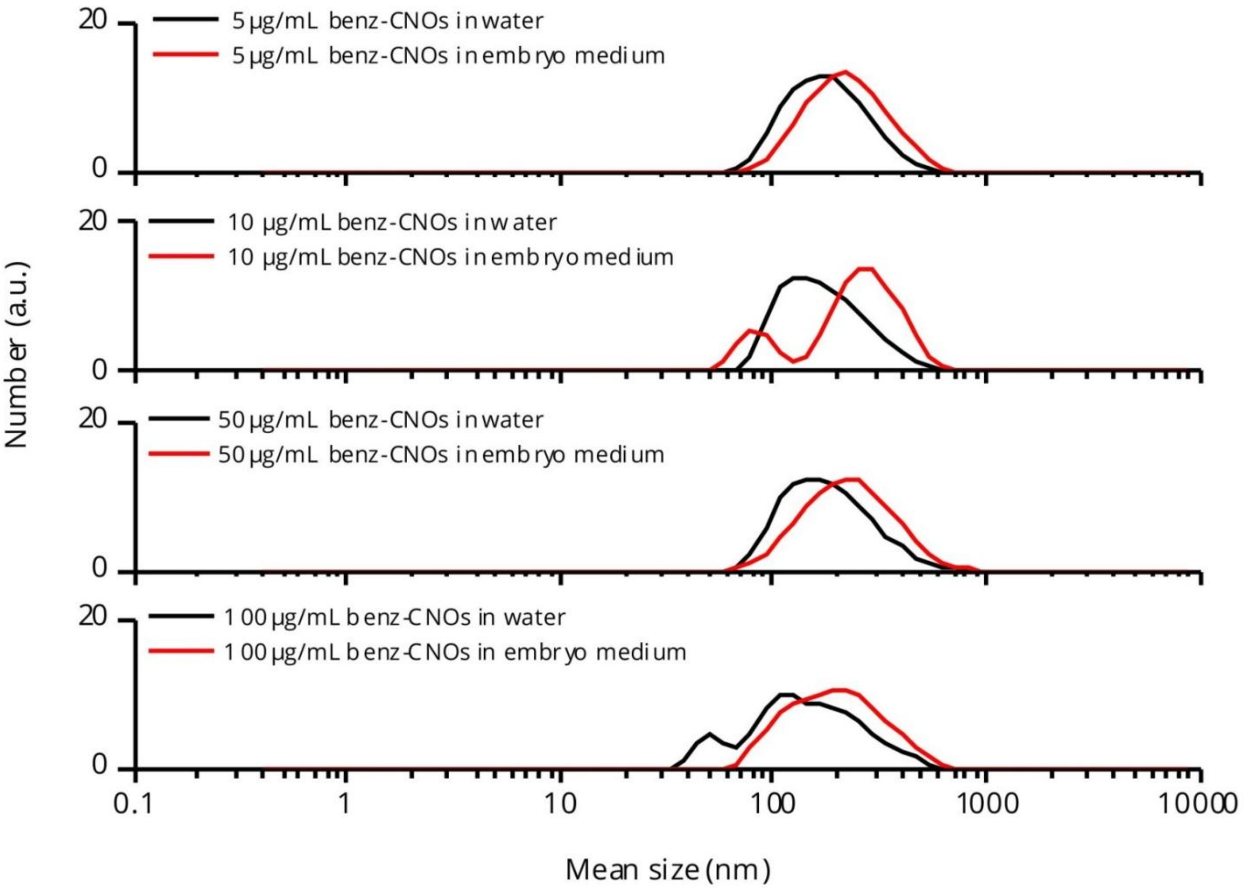

DLS measurements by number for benz-CNOs in water and embryo medium at a concentration of 5, 10, 50 and 100 µg mL^{-1}, respectively.

Table S1. ***Benz-CNOs diameter values obtained by DLS measurements***

[benz-CNOs] (µg/mL)	Diameter in water (nm)	Number Percentage in water (%)	Diameter in embryo medium (nm)	Number Percentage in embryo medium (%)
5	191± 83	100	233 ± 99	100
10	186 ± 93	100	85 ± 16<br>288 ± 81	18.6<br>81.4
50	195 ± 93	100	343 ± 111	100
100	53 ± 9<br>180 ± 98	14.3<br>85.7	218 ± 112	100

Hydrodynamic diameter from DLS analyses, together with standard deviations, for benz-CNOs in water and embryo medium at a concentration of 5, 10, 50 and 100 µg mL^{-1}, respectively.

Figure S4. ***BODIPY-CNOs diameter distribution obtained by DLS measurements***

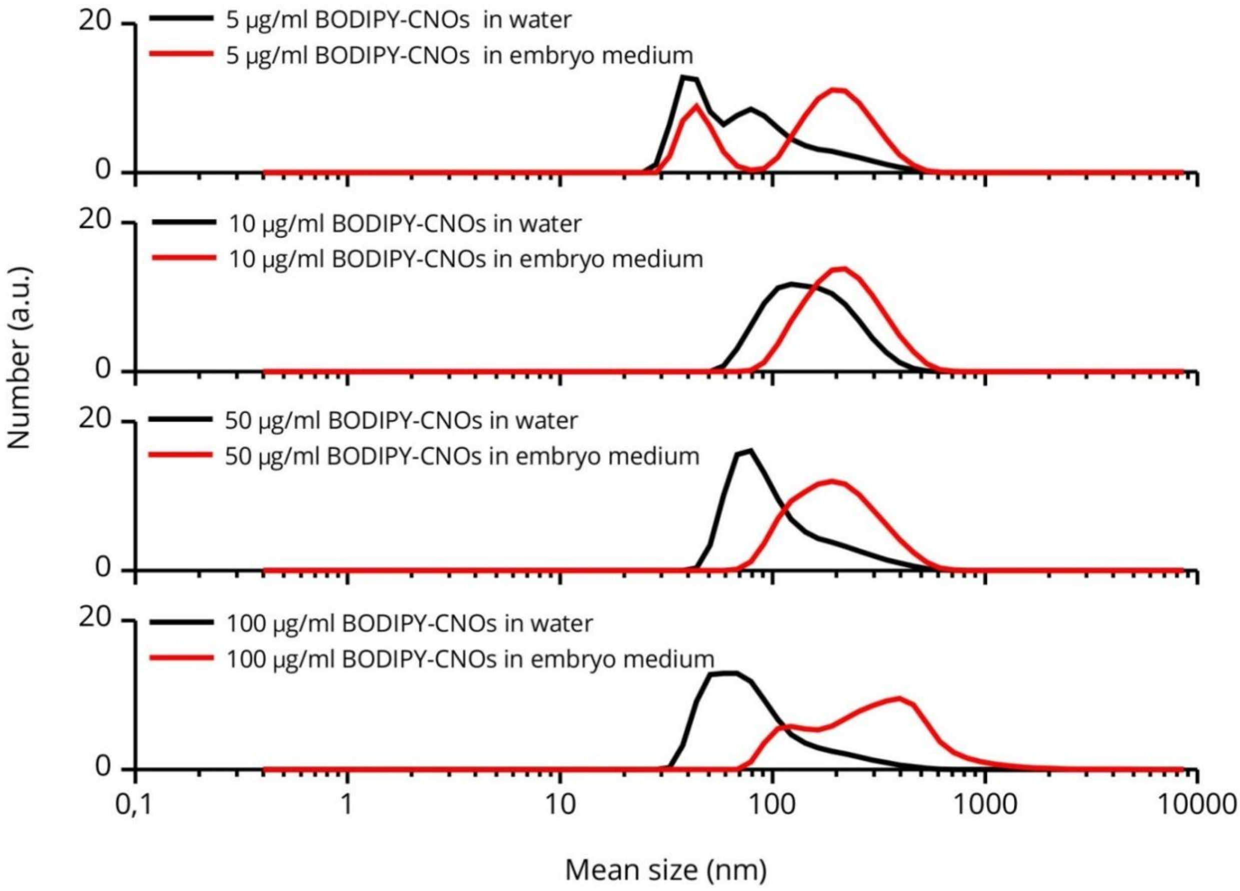

DLS measurements by number for BODIPY-CNOs in water and embryo medium at a concentration of 5, 10, 50 and 100 μg mL^{-1}, respectively.

Table S2. ***BODIPY-CNOs diameter values obtained by DLS measurements***

**[BODIPY-CNOs] (µg/mL)**	**Diameter in water (nm)**	**Number Percentage in water (%)**	**Diameter in embryo medium (nm)**	**Number Percentage in embryo medium (%)**
5	43 ± 8<br>128 ± 82	44.6<br>55.4	45 ± 8<br>218 ± 79	28.2<br>71.8
10	163 ± 75	100	229 ± 94	100
50	117 ± 79	100	214 ± 99	100
100	95 ± 71	100	125 ± 25<br>408 ± 309	25.2<br>74.8

Hydrodynamic diameter from DLS analyses, together with standard deviations, for BODIPY-CNOs in water and embryo medium at a concentration of 5, 10, 50 and 100 µg mL^{-1}, respectively.

Table S3. ***Benz-CNOs zeta potential values***

[benz-CNOs] (µg/mL)	Zeta potential in water (mV)	Percentage in water (%)	Zeta potential in embryo medium (mV)	Percentage in embryo medium (%)
5	-41 ± 5	100	-30 ± 5<br>-3 ± 2	99.6<br>0.4
10	-42 ± 5	100	-30 ± 5	100
50	-42 ± 4	100	-35 ± 5	100
100	-43 ± 5	100	-30 ± 4	100

Zeta potential values, together with standard deviations, for benz-CNOs in water and embryo medium at a concentration of 5, 10, 50 and 100 µg mL^{-1}, respectively.

Table S4. ***BODIPY-CNOs zeta potential values***

[BODIPY-CNOs] (µg/mL)	Zeta potential in water (mV)	Percentage in water (%)	Zeta potential in embryo medium (mV)	Percentage in embryo medium (%)
5	-36 ± 5	100	-31 ± 6	100
10	-34 ± 5	100	-32 ± 4	100
50	-29 ± 4	100	-30 ± 5	100
100	-23 ± 4	100	-24 ± 4	100

Zeta potential values, together with standard deviations, for BODIPY-CNOs in water and embryo medium at a concentration of 5, 10, 50 and 100 µg mL^{-1}, respectively.

Figure S5. Size statistical analysis on benz-CNOs and BODIPY-CNOs

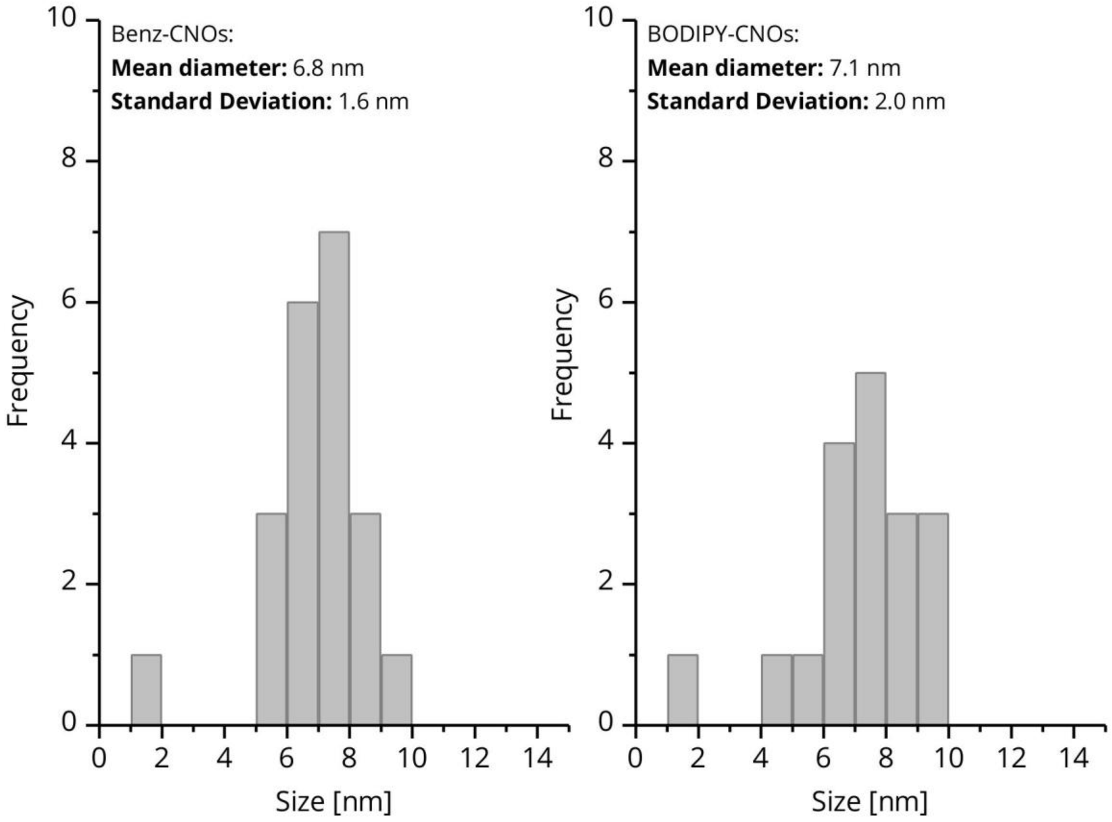

Size statistical analyses of benz-CNOs (left side) and BODIPY-CNOs (right side). The mean diameters and the standard deviations are indicated in the legend.

Figure S6. ***High magnification image of the zebrafish embryos and larvae represented in Figure 4.E, eye; YS, yolk sac; 48hpf, scale bars=1 mm.***

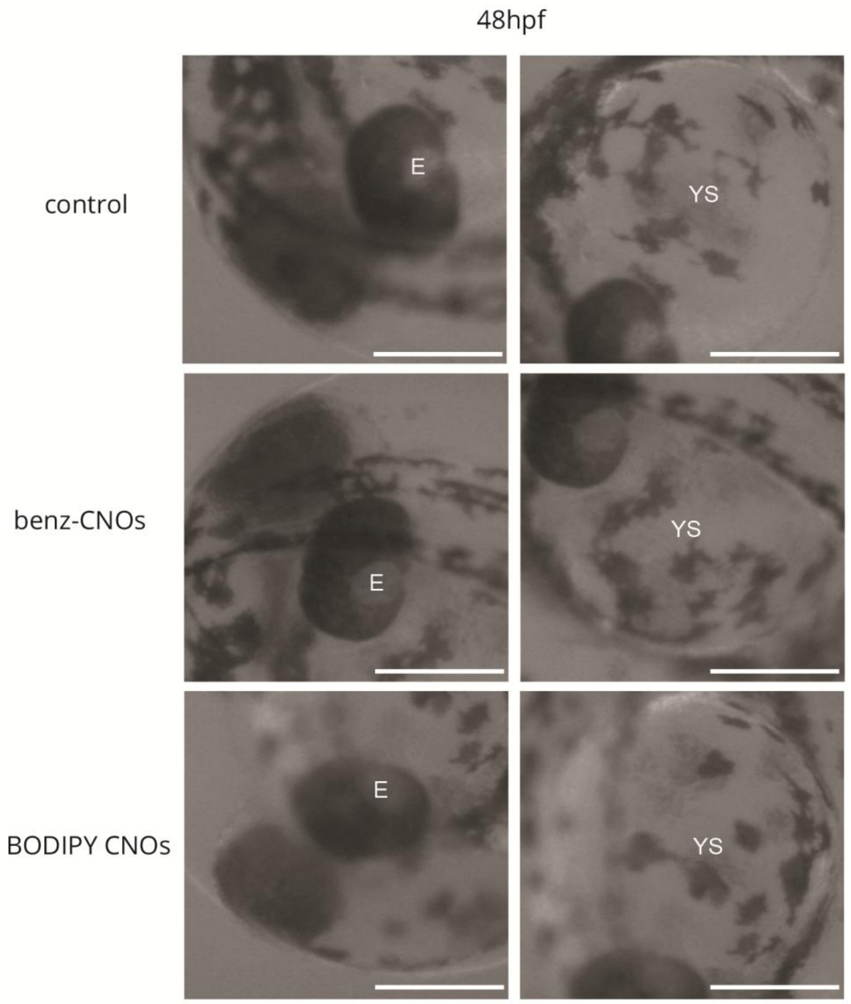

Figure S7. ***High magnification image of the zebrafish embryos and larvae represented in Figure 4. E, eye; YS, yolk sac. E, eye; YS, yolk sac; T, tail; F, finfold. 96hpf, scale bars=1 mm.***

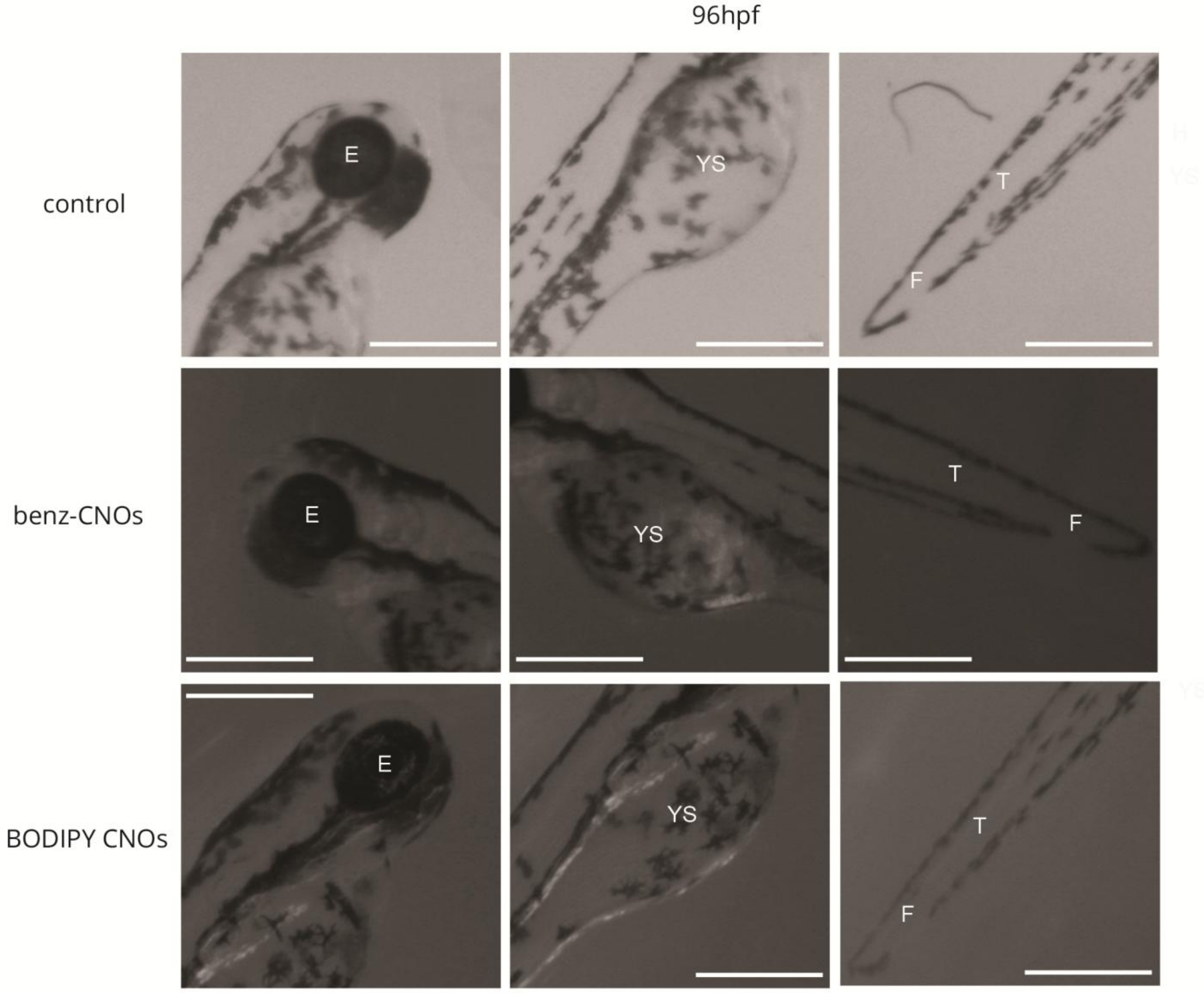

Figure S8. *Z-stack of Figure 6A. Step size= 21 µm. Scale bar= 100 µm.*

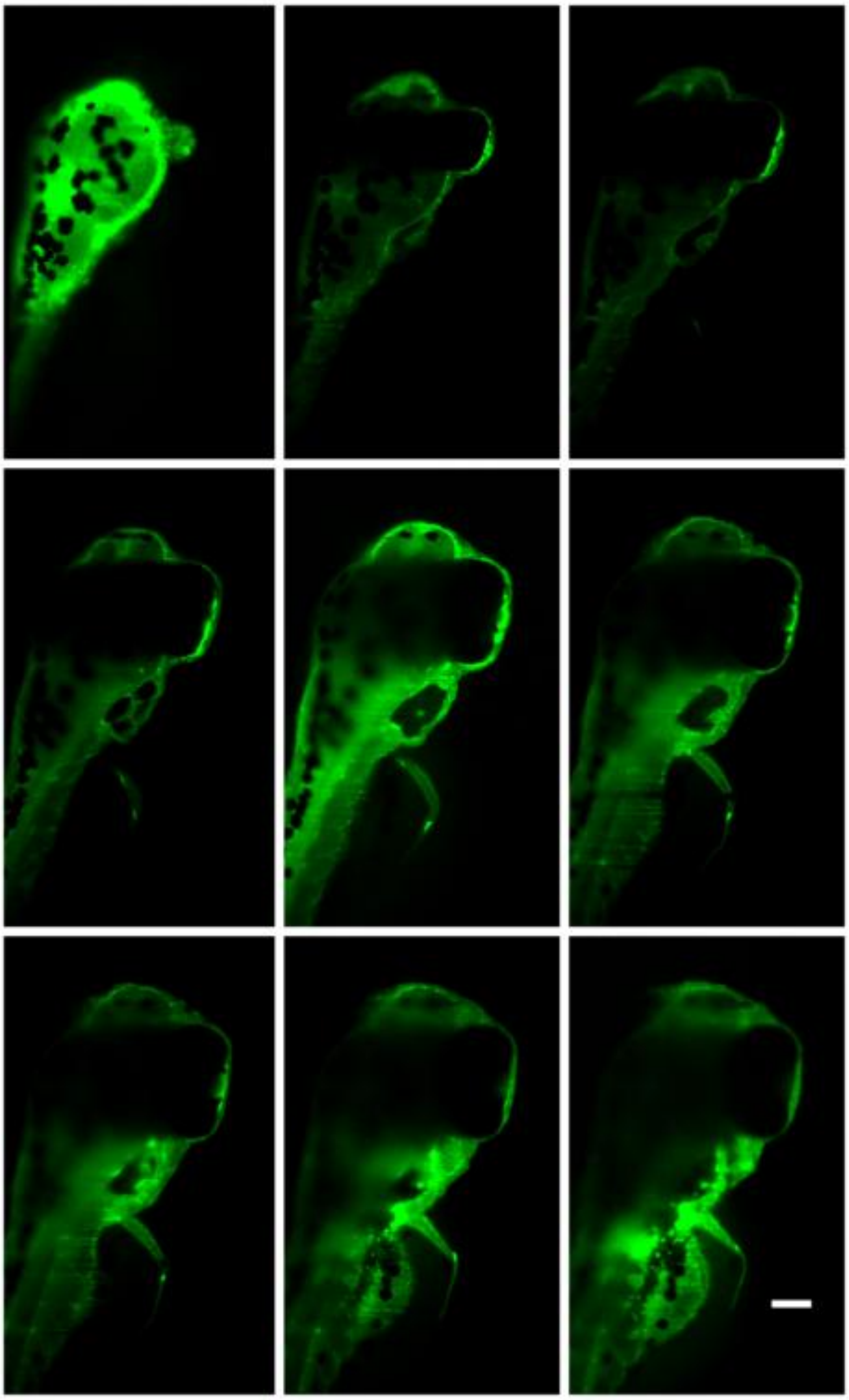

Figure S9. ***Z-stack of Figure 6B. Step size= 21 µm. Scale bar= 100 µm.***

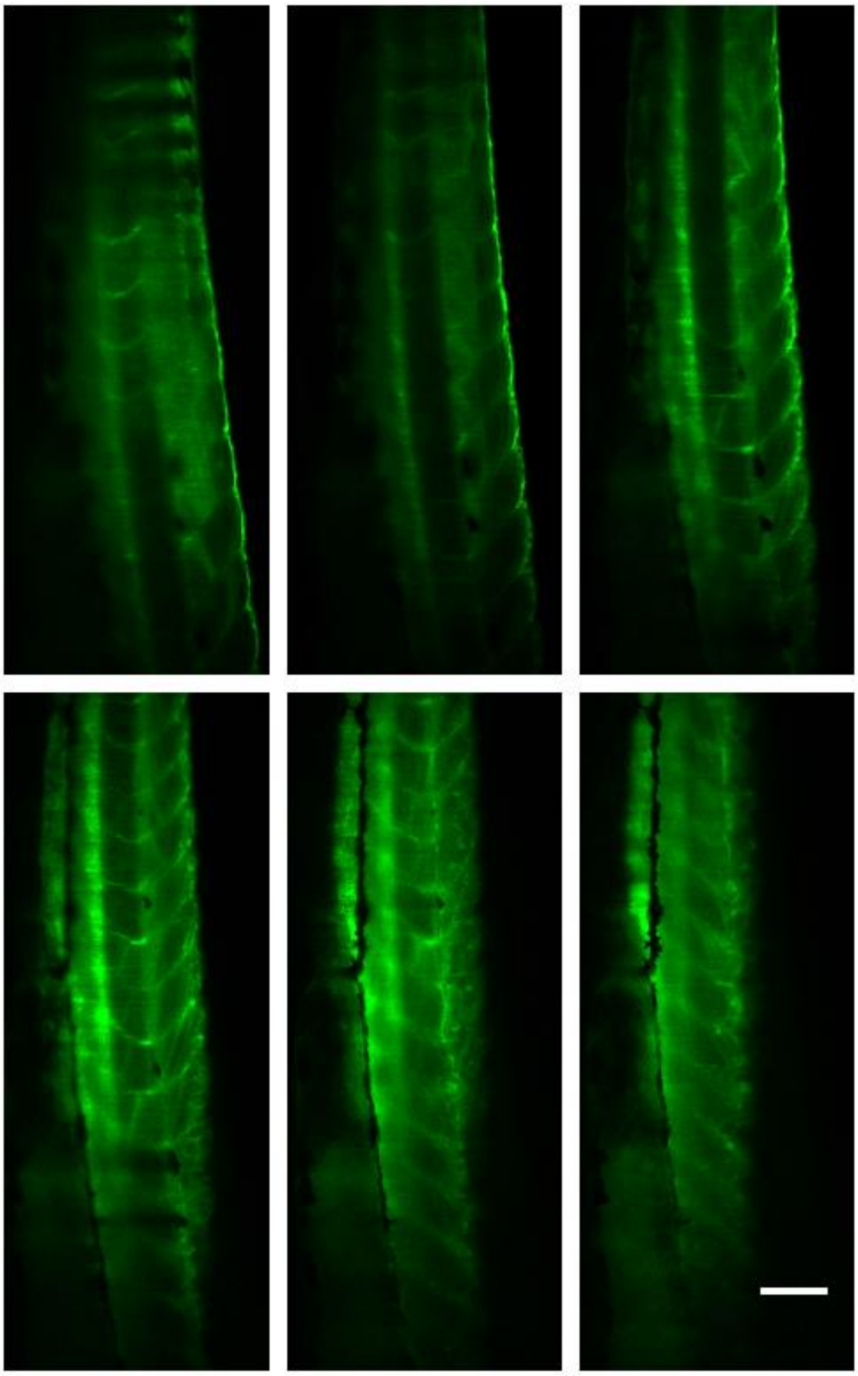

Supplement: Supplementary Information [file srep33923-s1.pdf]
